# Competition age: does it matter for swimmers?

**DOI:** 10.1186/s13104-022-05969-6

**Published:** 2022-02-23

**Authors:** Dennis-Peter Born, Ina Stäcker, Michael Romann, Thomas Stöggl

**Affiliations:** 1 Swiss Swimming Federation, Section for High-Performance Sports, Bern, Switzerland; 2 Department for Elite Sport, Swiss Federal Institute of Sport Magglingen, Hauptstrasse 247, 2532 Magglingen, Switzerland; 3 Department of Sport and Exercise Science, University of Salzburg, Salzburg, Austria; 4 Red Bull Athlete Performance Center, Salzburg, Austria

**Keywords:** Elite, Long-term athlete development, Reference data, Relative age, Swimming, Talent

## Abstract

**Objective:**

To establish reference data on required competition age regarding performance levels for both sexes, all swimming strokes, and race distances and to determine the effect of competition age on swimming performance in the context of other common age metrics. In total, 36,687,573 race times of 588,938 swimmers (age 14.2 ± 6.3 years) were analyzed. FINA (Fédération Internationale de Natation) points were calculated to compare race times between swimming strokes and race distances. The sum of all years of race participation determined competition age.

**Results:**

Across all events, swimmers reach top-elite level, i.e. > 900 FINA points, after approximately 8 years of competition participation. Multiple-linear regression analysis explained up to 40% of variance in the performance level and competition age showed a stable effect on all race distances for both sexes (*β* = 0.19 to 0.33). Increased race distance from 50 to 1500 m, decreased effects of chronological age (*β* = 0.48 to − 0.13) and increased relative age effects (*β* = 0.02 to 0.11). Reference data from the present study should be used to establish guidelines and set realistic goals for years of competition participation required to reach certain performance levels. Future studies need to analyze effects of transitions between various swimming strokes and race distances on peak performance.

**Supplementary Information:**

The online version contains supplementary material available at 10.1186/s13104-022-05969-6.

## Introduction

At international competitions, i.e. European championships, chronological age was related to success [1], possibly due to accumulated training time and competition experience, i.e. deliberate practice, contributing to the achievement of top-elite performance [2, 3]. As internationally successful swimmers reach peak performance between 21 and 26 years of age and peak performance duration is limited (2.6 ± 1.5 years) [4], swimmers may have to start early to reach top-elite performance on time and not to miss their window of opportunity.

While the concept of deliberate practice [2, 3] is hardly discussed among experts in the field, it may not apply to all sports to the same extent [5–7]. In some sports, athletes may require far less than the proposed 10 years of deliberate practice [2, 3] to achieve international success [7]. However, swimming is a highly technical sport that requires athletes to perform in a very specific and for mankind unusual environment. As water reduces movement efficiency compared to on-land locomotion [8], more practice in the specific environment may be required for top-elite success in swimming. Additionally, endurance sports, such as swimming, require a high aerobic capacity and benefit from accumulated hours of training over multiple years [9]. Therefore, the concept of deliberate practice may be more important in swimming compared to other sports and competition age may heavily contribute to elite-age success.

Additionally, the relative age, i.e. age-related difference between athletes born early and late in the year, affects talent selection and progression towards top-elite performance [10, 11]. By the age of 8 years, early compared to late in the year born individuals have a physical advantage of 10% [10]. Therefore, by the age of 13, twice the number of Australian national level swimmers were born early in the year [11]. The RAE was larger in male compared to female swimmers [12, 13] and most pronounced in short-distance events, butterfly (BU), and breaststroke (BR) [12]. The RAE was most evident in the younger age-categories and reversed towards senior age [12]. Therefore, the main aim of the study was to determine the effect of competition age, i.e. accumulated years of competition participation, on swimming performance in the context of the other common age metrics, i.e. relative and chronological age. Additionally, reference data were established for competition age required to reach various performance levels across both sexes, all swimming strokes, and all race distances.

## Main text

### Materials and methods

#### Subjects

In total, 36,687,573 race times of 588,938 swimmers (males: 15.9 ± 5.8 and females: 14.7 ± 4.7 years of age) from the years 2000 until 2019 were included in the present study. Data were provided from the official database [14] of the European Swimming Federation LEN (Ligue Européenne de Natation) with permission for anonymized publication of the results. The study was approved by the lead institution’s internal review board for ethical affairs (Reg.-Nr. 139_LSP_V01) and is in accordance with the latest version of the code of conduct of the World Medical Association for studies involving human subjects (WMA Declaration of Helsinki). No consent for participation was required as anonymized race results were provided from a publicly available database and analyzed ex post facto.

#### Data analysis

To compare race times between swimming strokes and race distances, FINA (Fédération Internationale de Natation) points for each race time were calculated [15]. FINA points are the official method of the International Swimming Association to relate race times to the current world record, i.e. 1000 points.

$$\mathrm{FINA\,points }\,[\mathrm{a}.\mathrm{u}.]=1000 \times {\left(\frac{\mathrm{World\,record}}{\mathrm{Race\,time}}\right)}^{3}$$

*Analysis step 01* From the 2019 race data, each individual swimmer’s best race, i.e. most FINA points, was used to establish the ranking for each race category. As race categories were defined: (a) all events including all swimming strokes and all race distances from 50 to 1500 m; (b) each swimming stroke, i.e. BU, backstroke (BA), BR, freestyle (FR), individual medley (IM), using pooled data of its 50, 100, and 200 m races; (c) each race distance using the 50, 100, 200, 400, 800, and 1500 m FR events. The rankings were established based on long-course races, due to their superior recognition, i.e. at the Olympics, compared to short-course events (25 m pool length).

*Analysis step 02* Within each race category, each swimmer was tracked retrospectively, and the races extracted for every year from 2000 to 2019. Total number of years with race times of the particular race category determined competition age. As short-course races contribute to the training and development process, short- and long-course races were included for determination of competition age (Additional file 1: Fig. S1). The analysis was performed for male and female swimmers individually. Data processing was performed with Python [16] using the ‘pandas’ library [17] and Microsoft Excel (Excel 2016, Microsoft Corporation, Redmond, WA, USA).

#### Statistical analysis

Participants aged beyond the mean ± three standard deviations of the race category were removed as outliers [18]. Normality was investigated with standardized residuals showing a random pattern across predicted values in the scatter plot, a Gaussian distribution in the histogram, and a straight diagonal line in the Q-Q plot [18]. Multiple linear regression analysis was used to assess the effect of competition age [years] in the context of the other common age metrics, i.e. relative [month of birth] and chronological age [years]. Swimming performance, i.e. FINA points, were used as dependent variable. Collinearity was controlled with a tolerance > 0.10 and a variance inflation factor < 10 [18]. An alpha-level < 0.05 indicated a significant effect. The statistical analysis was performed using JASP statistical software package version 0.14 (JASP-Team, University of Amsterdam, Amsterdam, The Netherlands).

### Results

Across all events, swimmers that reached a performance level of > 900 FINA points accumulated approximately 8 years of competition practice (males 7.7 ± 4.2 years and females 8.0 ± 3.2 years). Table 1 shows the descriptive analysis of competition age for different levels of swimming performance.

**Table 1:** Descriptive analysis of competition age (mean ± standard deviation) required to reach various performance levels based on FINA points

	FINA points									
	1000–900	900–800	800–700	700–600	600–500	500–400	400–300	300–200	200–100	100–0
Males										
Subjects [n]	155	2167	8490	22,247	40,894	51,045	53,351	55,757	52,577	20,164
Chronological age [years]	23.5 ± 3.6	21.7 ± 3.3	20.1 ± 3.2	18.8 ± 3.4	17.7 ± 3.8	17.1 ± 4.9	16.3 ± 5.9	15.1 ± 6.3	14.3 ± 6.1	14.9 ± 7.0
Competition age [years]										
All events	7.7 ± 4.2	6.6 ± 3.8	5.2 ± 3.7	4.5 ± 3.4	4.2 ± 3.3	3.8 ± 2.9	3.0 ± 2.3	2.3 ± 1.6	1.8 ± 1.1	1.4 ± 0.9
Butterfly	10.0 ± 3.7	7.4 ± 4.0	6.4 ± 3.9	6.0 ± 3.9	5.7 ± 3.7	5.3 ± 3.3	4.5 ± 2.7	3.6 ± 2.1	2.8 ± 1.6	2.4 ± 1.4
Backstroke	8.9 ± 4.1	7.1 ± 3.8	5.2 ± 3.7	5.2 ± 3.8	5.0 ± 3.6	4.7 ± 3.3	4.0 ± 2.6	3.0 ± 1.9	2.1 ± 1.3	1.5 ± 0.9
Breaststroke	6.2 ± 3.0	7.2 ± 4.0	5.9 ± 4.0	5.4 ± 3.9	5.1 ± 3.6	4.8 ± 3.4	4.2 ± 2.8	3.1 ± 2.1	2.1 ± 1.4	1.6 ± 1.1
Freestyle	8.9 ± 4.1	7.0 ± 4.1	5.9 ± 3.9	5.1 ± 3.6	4.7 ± 3.4	4.3 ± 3.1	3.2 ± 2.3	2.4 ± 1.5	1.8 ± 1.2	1.4 ± 0.9
Individual medley	9.3 ± 6.1	7.7 ± 4.1	6.3 ± 4.0	5.6 ± 3.7	5.2 ± 3.3	5.0 ± 3.0	4.1 ± 2.4	3.1 ± 1.8	2.3 ± 1.3	2.4 ± 1.7
50 m Freestyle	13.0 ± 0.0	6.8 ± 3.7	6.3 ± 4.0	5.8 ± 3.9	5.4 ± 3.7	5.0 ± 3.2	3.9 ± 2.5	3.0 ± 1.7	2.3 ± 1.3	2.0 ± 1.1
100 m Freestyle	8.8 ± 3.8	7.3 ± 4.2	6.2 ± 4.0	5.5 ± 3.6	5.0 ± 3.4	4.5 ± 2.9	3.4 ± 2.2	2.6 ± 1.6	2.0 ± 1.2	1.6 ± 1.0
200 m Freestyle	7.7 ± 5.0	7.0 ± 4.0	6.5 ± 4.0	5.5 ± 3.7	5.0 ± 3.3	4.8 ± 2.9	3.8 ± 2.3	2.9 ± 1.7	2.3 ± 1.4	1.9 ± 1.4
400 m Freestyle	7.7 ± 4.0	6.4 ± 3.7	5.5 ± 3.9	4.9 ± 3.3	4.9 ± 3.3	4.7 ± 2.8	4.0 ± 2.5	3.1 ± 2.1	2.6 ± 1.6	2.6 ± 2.3
800 m Freestyle	7.6 ± 5.6	7.3 ± 3.9	5.5 ± 3.6	5.4 ± 3.5	4.9 ± 3.3	4.9 ± 2.8	4.5 ± 2.8	3.7 ± 2.5	3.3 ± 2.4	3.2 ± 2.2
1500 m Freestyle	9.2 ± 4.7	6.5 ± 3.7	5.6 ± 3.9	5.2 ± 3.4	5.0 ± 3.0	4.9 ± 2.8	4.3 ± 2.7	3.5 ± 2.4	3.1 ± 2.2	2.9 ± 0.9
Females										
Subjects [n]	111	1365	7026	19,673	39,591	54,373	58,562	54,930	37,143	9317
Chronological age [years]	21.4 ± 2.7	20.9 ± 3.8	19.1 ± 3.2	17.3 ± 2.9	16.2 ± 2.9	15.5 ± 3.7	15.0 ± 4.8	14.2 ± 5.6	14.0 ± 6.1	14.8 ± 7.3
Competition age [years]										
All events	8.0 ± 3.2	6.8 ± 3.9	5.0 ± 3.3	4.3 ± 3.2	3.9 ± 3.0	3.7 ± 2.7	3.0 ± 2.2	2.2 ± 1.5	1.6 ± 1.0	1.4 ± 0.8
Butterfly	11.0 ± 4.6	7.0 ± 4.2	6.0 ± 3.6	5.6 ± 3.7	5.3 ± 3.4	5.0 ± 3.0	4.3 ± 2.6	3.3 ± 2.1	2.4 ± 1.5	1.9 ± 1.3
Backstroke	7.4 ± 2.7	7.2 ± 3.9	5.5 ± 3.6	4.8 ± 3.6	4.5 ± 3.3	4.3 ± 2.9	3.7 ± 2.4	2.7 ± 1.8	1.8 ± 1.2	1.5 ± 1.0
Breaststroke	9.4 ± 3.8	7.3 ± 3.8	5.6 ± 3.7	4.8 ± 3.6	4.5 ± 3.3	4.3 ± 3.0	3.9 ± 2.5	2.7 ± 1.9	1.8 ± 1.3	1.5 ± 1.0
Freestyle	8.6 ± 3.1	7.2 ± 4.0	5.7 ± 3.7	4.7 ± 3.4	4.4 ± 3.1	4.2 ± 2.8	3.3 ± 2.2	2.3 ± 1.6	1.7 ± 1.1	1.4 ± 0.9
Individual medley	8.4 ± 1.9	7.5 ± 3.9	5.8 ± 3.6	5.2 ± 3.5	5.0 ± 3.2	4.7 ± 2.7	3.8 ± 2.3	2.8 ± 1.7	2.3 ± 1.4	2.7 ± 2.4
50 m Freestyle	8.3 ± 2.6	8.3 ± 4.7	6.1 ± 4.1	5.2 ± 3.6	5.0 ± 3.3	4.6 ± 2.8	3.5 ± 2.2	2.4 ± 1.5	1.7 ± 1.1	1.4 ± 1.0
100 m Freestyle	8.8 ± 1.9	8.0 ± 4.4	6.0 ± 3.7	5.3 ± 3.5	4.8 ± 3.2	4.5 ± 2.8	3.5 ± 2.2	2.6 ± 1.7	1.9 ± 1.2	1.6 ± 1.1
200 m Freestyle	10.3 ± 3.7	7.4 ± 3.9	6.0 ± 3.6	5.1 ± 3.4	4.9 ± 3.1	4.6 ± 2.7	3.7 ± 2.2	2.8 ± 1.8	2.2 ± 1.3	2.1 ± 1.5
400 m Freestyle	4.0 ± 1.4	7.6 ± 4.2	6.1 ± 3.8	5.2 ± 3.3	4.9 ± 3.1	4.6 ± 2.7	4.1 ± 2.4	3.2 ± 2.0	2.6 ± 1.8	3.2 ± 2.7
800 m Freestyle	7.3 ± 3.5	7.1 ± 4.3	6.4 ± 4.0	5.2 ± 3.4	5.3 ± 3.1	4.9 ± 2.7	4.5 ± 2.5	3.7 ± 2.4	3.4 ± 2.8	2.8 ± 1.7
1500 m Freestyle	8.5 ± 6.2	8.8 ± 4.5	7.0 ± 3.8	6.1 ± 3.5	5.9 ± 3.3	5.7 ± 2.8	5.7 ± 2.7	5.0 ± 2.9	4.0 ± 2.2	2.6 ± 1.6

Regression analysis explained up to 40% of variance in swimming performance, i.e. FINA points, and showed a significant effect of competition age, chronological age, and relative age (*P* < 0.001). Regarding race distances, there was a stable effect of competition age from 50 to 1500 m in males (*β* = 0.21 to 0.33, Table 2) and females (*β* = 0.19 to 0.33, Table 3). However, the effect of chronological age decreased the longer the race distance in males (*β* = 0.48 to − 0.12) and females (*β* = 0.39 to − 0.13). The effect of relative age was fairly small yet increased with race distance from 50 to 1500 m in males (*β* = 0.02 to 0.11) and females (*β* = 0.03 to 0.08).

**Table 2:** Multiple linear regression analysis of swimming performance, i.e. FINA points, as dependent variable and competition (comp.) [years] age, relative age [month of birth], and chronological (chronol.) [years] age as predictors with standardized (beta_i) and unstandardized (b_i) regression coefficients in male swimmers

Regression model					Regression coefficients			
n	*R*^*2*^	*F*	*p*		beta_i	b_i	t_i	*p*
All events				Comp. age	0.34	24.6	74	< 0.001
39,487	0.25	*F*_(3|39483)_ = 4326	< 0.001	Relative age	0.08	4.2	18	< 0.001
				Chronol. age	0.24	7.0	52	< 0.001
Butterfly				Comp. age	0.24	14.3	54	< 0.001
42,926	0.29	*F*_(3|42922)_ = 5897	< 0.001	Relative age	0.03	1.6	8	< 0.001
				Chronol. age	0.39	13.0	86	< 0.001
Backstroke				Comp. age	0.24	15.5	39	< 0.001
23,603	0.36	*F*_(3|23599)_ = 4400	< 0.001	Relative age	0.04	1.8	7	< 0.001
				Chronol. age	0.44	16.7	72	< 0.001
Breaststroke				Comp. age	0.28	17.5	46	< 0.001
22,258	0.34	*F*_(3|22254)_ = 3878	< 0.001	Relative age	0.04	1.8	7	< 0.001
				Chronol. age	0.40	13.4	66	< 0.001
Freestyle				Comp. age	0.33	22.0	69	< 0.001
34,759	0.32	*F*_(3|34755)_ = 5502	< 0.001	Relative age	0.05	2.5	11	< 0.001
				Chronol. age	0.34	10.9	70	< 0.001
Individual medley				Comp. age	0.14	7.7	18	< 0.001
16,070	0.33	*F*_(3|16066)_ = 2635	< 0.001	Relative age	0.04	1.8	6	< 0.001
				Chronol. age	0.48	20.4	63	< 0.001
50 m Freestyle				Comp. age	0.33	19.7	85	< 0.001
52,043	0.30	*F*_(3|52039)_ = 7304	< 0.001	Relative age	0.03	1.5	9	< 0.001
				Chronol. age	0.32	8.1	82	< 0.001
100 m Freestyle				Comp. age	0.24	15.6	45	< 0.001
28,309	0.40	*F*_(3|28305)_ = 6390	< 0.001	Relative age	0.02	0.9	4	< 0.001
				Chronol. age	0.48	20.0	89	< 0.001
200 m Freestyle				Comp. age	0.21	12.6	32	< 0.001
20,225	0.33	*F*_(3|20221)_ = 3279	< 0.001	Relative age	0.03	1.5	6	< 0.001
				Chronol. age	0.43	18.3	65	< 0.001
400 m Freestyle				Comp. age	0.26	15.0	31	< 0.001
14,005	0.11	*F*_(3|14001)_ = 570	< 0.001	Relative age	0.09	4.0	11	< 0.001
				Chronol. age	0.11	3.3	14	< 0.001
800 m Freestyle				Comp. age	0.23	11.8	20	< 0.001
7695	0.07	*F*_(3|7691)_ = 195	< 0.001	Relative age	0.11	4.8	10	< 0.001
				Chronol. age	− 0.12	− 2.4	− 11	< 0.001
1500 m Freestyle				Comp. age	0.22	11.5	17	< 0.001
5466	0.07	*F*_(3|5462)_ = 130	< 0.001	Relative age	0.11	4.8	9	< 0.001
				Chronol. age	− 0.10	− 2.3	− 7	< 0.001

**Table 3:** Multiple linear regression analysis of swimming performance, i.e. FINA points, as dependent variable and competition (comp.) age [years], relative age [month of birth], and chronological (chronol.) age [years] as predictors with standardized (beta_i) and unstandardized (b_i) regression coefficients in female swimmers

Regression model					Regression coefficients			
n	*R*^*2*^	*F*	*p*		beta_i	b_i	t_i	*p*
All events				Comp. age	0.34	22.9	71	< 0.001
39,572	0.20	*F*_(3|39568)_ = 3361	< 0.001	Relative age	0.09	4.5	21	< 0.001
				Chronol. age	0.18	5.3	37	< 0.001
Butterfly				Comp. age	0.21	12.2	35	< 0.001
26,925	0.28	*F*_(3|26921)_ = 3436	< 0.001	Relative age	0.04	1.9	8	< 0.001
				Chronol. age	0.38	16.3	63	< 0.001
Backstroke				Comp. age	0.25	15.9	42	< 0.001
26,136	0.28	*F*_(3|26132)_ = 3456	< 0.001	Relative age	0.06	2.4	10	< 0.001
				Chronol. age	0.35	14.0	58	< 0.001
Breaststroke				Comp. age	0.28	17.0	45	< 0.001
23,676	0.26	*F*_(3|23672)_ = 2754	< 0.001	Relative age	0.04	1.8	8	< 0.001
				Chronol. age	0.31	10.8	50	< 0.001
Freestyle				Comp. age	0.32	20.3	64	< 0.001
36,037	0.26	*F*_(3|36033)_ = 4184	< 0.001	Relative age	0.06	2.8	13	< 0.001
				Chronol. age	0.27	9.3	54	< 0.001
Individual medley				Comp. age	0.14	7.4	18	< 0.001
18,263	0.25	*F*_(3|18259)_ = 2070	< 0.001	Relative age	0.06	2.4	10	< 0.001
				Chronol. age	0.40	17.4	53	< 0.001
50 m Freestyle				Comp. age	0.33	18.6	62	< 0.001
31,906	0.29	*F*_(3|31902)_ = 4319	< 0.001	Relative age	0.03	1.1	5	< 0.001
				Chronol. age	0.30	10.2	56	< 0.001
100 m Freestyle				Comp. age	0.24	14.2	42	< 0.001
29,808	0.32	*F*_(3|29804)_ = 4583	< 0.001	Relative age	0.04	1.6	7	< 0.001
				Chronol. age	0.39	16.6	69	< 0.001
200 m Freestyle				Comp. age	0.20	11.4	28	< 0.001
21,365	0.26	*F*_(3|21361)_ = 2431	< 0.001	Relative age	0.05	2.2	8	< 0.001
				Chronol. age	0.37	16.1	53	< 0.001
400 m Freestyle				Comp. age	0.26	14.4	32	< 0.001
14,646	0.11	*F*_(3|14642)_ = 595	< 0.001	Relative age	0.08	3.3	10	< 0.001
				Chronol. age	0.10	3.0	12	< 0.001
800 m Freestyle				Comp. age	0.25	12.4	22	< 0.001
8104	0.07	*F*_(3|8100)_ = 215	< 0.001	Relative age	0.08	3.3	8	< 0.001
				Chronol. age	− 0.13	− 2.7	− 12	< 0.001
1500 m Freestyle				Comp. age	0.19	8.8	14	< 0.001
5654	0.05	*F*_(3|5650)_ = 89	< 0.001	Relative age	0.06	2.4	5	< 0.001
				Chronol. age	− 0.13	− 3.3	− 10	< 0.001

### Discussion

Swimmers accumulated approximately 8 years of competition practice to reach top-elite level, i.e. > 900 FINA points, regardless of event. The present descriptive data show years of competition practice needed to reach various performance levels for each swimming stroke and race distance. While the regression model explained up to 40% of variance in swimming performance depending on the event, the effect of competition age remained stable across all race distances. The effect of chronological age continuously decreased, and the effect of relative age continuously increased the longer the race distance, i.e. from 50 to 1500 m.

Previous studies showed the largest relative age effect at early junior age that decreased the older the swimmers [10] and even reversed towards senior age [12]. As the present study analyzed swimming performance up to elite age, the before mentioned aspect is one explanation as to why the relative age effect was comparatively smaller in the regression model than the other age metrics. With a particular interest on the RAE and potential differences between swimming strokes and sexes [12, 13], studies should focus on the young age groups from which the RAE originates [10, 11]. Previous studies found the largest RAE in short-distance swimming events [12]. With the large age-range analyzed here and age of peak performance being lowest in the longer swimming events [4], the present study showed an opposite trend towards an increasing RAE the longer the race distance. In conclusion, coaches and federation officials should be aware of the relative age effect. Early deselection of young swimmers with a late birthday in the year results in an irreversible loss of talents [11].

The present study showed a stable effect of competition age for all swimming events, which supports previous findings that top-elite performance needs to be developed over time and requires accumulated years of practice [2, 3]. However, as swimmers usually enter competitive swimming aged 8–10 years, and age of peak performance is 21–26 years [4], the average 8 years of accumulated competition practice necessary to reach top-elite performance (> 900 FINA points) still allow enough time for solid and progressive talent development. This is particularly important to lay the foundation for progression in the flat part of the performance curve towards elite age [19], when swimmers reach a performance level of > 900 FINA points. In this regard, talent programs benefit from a less hasty performance progression, a less harsh selection process, and increased focus on long-term performance development rather than short-term success during adolescence [20–22]. As ‘lack of enjoyment’ and ‘getting bored’ are two major factors for drop-out from swimming [23], particular attention should be placed on keeping swimmers in the system and motivated for training and competition [24].

Previous studies show the value of deliberate play which is the unspecific and unstructured involvement in the same or other sports [25, 26]. Compared to their national counterparts, world class athletes are in general more involved in other sports [6]. Due to the specific characteristics of water [8], swimmers may have to accumulate a larger volume of training and competition in their sport-specific environment compared to other sports. Thus, other aquatic sports, such as water polo, underwater rugby, or competitive aquatic lifesaving, may contribute to the development of water feeling, swimming technique, aerobic, and anaerobic endurance, while maintaining a high level of enjoyment [27, 28]. Additionally, recent studies showed the importance of start and turn performances for modern swim races [29, 30]. The push-off from a solid base, i.e. starting block and pool wall, requires high leg strength and power [31, 32]. Therefore, swimmers could also benefit from on-land activities and weight-bearing sports more than traditionally expected. From a practical perspective, coaches and federation officials should be aware of the concept of deliberate play [25, 26]. Promoting other aquatic and on-land sports during adolescence helps to maximize volume of practice, while sustaining enjoyment and motivation to keep young swimmers in the system rather than losing them to other endurance or explosive sports [23].

### Conclusion

The stable effect of competition age across all swimming events shown in the present study supports the findings that accumulated practice contributes to elite age success [2, 3], in particular in highly technical endurance sports, such as swimming. The approximately 8 years of accumulated competition practice required to reach top-elite performance (> 900 FINA points) still allows enough time to build a solid foundation with broad and variable skill acquisition before reaching peak performance age. Reference data from the present study should be used to establish guidelines and set realistic goals for years of competition practice required to reach various performance levels.

## Limitations

The present study determined competition age, assuming that regular competition participation is part of a well-structured training process. However, future studies should use questionnaires and training diaries to determine training age. FINA point ranking was established based on each swimmer’s best race within the race category. As swimmers specialize in a particular swimming stroke rather than race distance [33], the swimming strokes were compared using pooled data of the 50, 100, and 200 m events. Therefore, transitions between race distances were not accounted for. As 800 m (for males) and 1500 m (for females) were added to the 2021 Olympic program [34], long-distance swimmers may have started to compete in both events with high success rates despite low competition age in one of the events. Future studies should pay particular attention to transitions and cross effects between swimming strokes and race distances. Additionally, for top-elite swimmers, there may be a non-linear relationship between years of practice and performance level. After an initial steep incline, the curve would be expected to plateau and increased number of years in competition would not further translate into improved swimming performance. With the large sample size analyzed in the present study including swimmers from various performance levels down to < 100 FINA points, such relationship was not found. Future studies should investigate individual career pathways and competition history towards top-elite success based on longitudinal tracking.

## Supplementary Information

**Additional file 1: Figure S1.** Flow chart of the data analysis procedure.

## Data Availability

Data are available on request by the corresponding author.

## Abbreviations

FINA: Fédération Internationale de Natation
LEN: Ligue Européenne de Natation
WMA: World Medical Association
BU: Butterfly
BA: Backstroke
BR: Breaststroke
FR: Freestyle
IM: Individual medley
