# Supplementary figures and images for: Competition age: does it matter for swimmers?
Source: BMC Res Notes. 2022 Feb 23;15:82. doi: 10.1186/s13104-022-05969-6 (PMC8867847; doi:10.1186/s13104-022-05969-6)

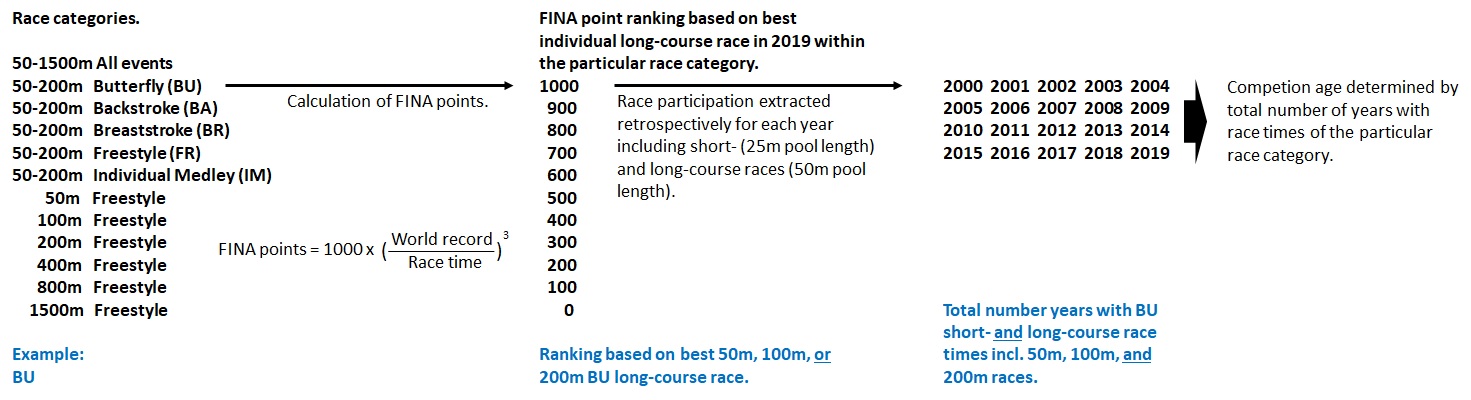

Supplement: Supplementary file 1 — Additional file 1: Figure S1. Flow chart of the data analysis procedure. [file 13104_2022_5969_MOESM1_ESM.jpg]
